# Perceived Cognition after Percutaneous Coronary Intervention: Association with Quality of Life, Mood and Fatigue in the THORESCI Study

**DOI:** 10.1007/s12529-016-9624-1

**Published:** 2016-12-28

**Authors:** Stefanie Duijndam, Johan Denollet, Ivan Nyklíček, Nina Kupper

**Affiliations:** Department of Medical and Clinical Psychology, CoRPS—Centre of Research on Psychological and Somatic Diseases, Tilburg University, Tilburg, the Netherlands

**Keywords:** Age, Cognition, Fatigue, Mood, Percutaneous coronary intervention, Quality of life

## Abstract

**Purpose:**

Percutaneous coronary intervention (PCI) is a common invasive procedure for the treatment of coronary artery diseases. Long-term cognitive functioning after PCI and its association with health-related quality of life (HRQL) and psychological factors is relatively unknown. The aim of this study is to examine whether perceived cognitive functioning during the year after PCI is associated with HRQL over this time period, and whether mood, fatigue, and age are associated with changes in perceived cognition and HRQL.

**Methods:**

Patients undergoing PCI (*n* = 384, 79% male, mean age = 63, SD = 10) were recruited in the observational *Tilburg Health Outcome Registry of Emotional Stress after Coronary Intervention (THORESCI)* cohort study. Perceived concentration and attention problems, HRQL, mood, and fatigue were assessed at baseline, at 1-month and 12-month follow-up.

**Results:**

General linear mixed modeling analysis showed that across time, between- and within-subject differences in perceived concentration problems were associated with a reduced HRQL in all domains independent of clinical and demographic covariates. Only a part of this association could be explained by negative mood, fatigue, and older age. Similar findings were found for between-subject differences in perceived attention problems.

**Conclusions:**

Between-subject differences and within-subject changes in perceived cognition in PCI patients were strongly associated with HRQL across time, such that poorer perceived cognition was associated with poorer HRQL, independent of demographic and clinical variables. Most of the associations were also independent of mood and fatigue. The results should increase the awareness of clinicians for the role of cognition in the cardiac rehabilitation and recovery post-PCI.

## Introduction

Health-related quality of life (HRQL) is considered an important indicator of health outcome after percutaneous coronary intervention (PCI) [1, 2]. Greater severity of coronary artery diseases (CAD) [3], symptoms of depression and fatigue [4], and reduced cognitive functioning [5] have been related to decreased HRQL.

Cognitive functioning is an intellectual process by which one becomes aware of, perceives, or comprehends ideas, and it involves mental processes such as perception, thinking, reasoning, and remembering [6]. Research has focused on the cognitive effects of carotid artery treatments [7, 8], but less is known about these effects following PCI [9–11] or coronary artery bypass graft (CABG) [9–13] with inconsistent findings, varying from no to severe impairments in concentration, attention, memory , and psychomotor speed after treatment [12–14]**.** Some studies have examined cognitive problems after CABG as compared to PCI 1 year after treatment [10, 11, 13, 15]. Because PCI patients were not examined as the main study group in these studies, the current study will investigate the extent and course of cognitive complaints after PCI.

Notwithstanding well-known benefits, neuropsychological testing to assess cognitive function often is not feasible or affordable in clinical settings [16]. In recent years studies (e.g., [16]) have been advocating the use of self-reported perceived cognitive function as a screening and stratification tool. This is supported by studies showing that perceived cognitive function may predict changes in brain function and may precede more overt deficits [17].

Both perceived and neuropsychologically assessed cognitive functioning is found to be a major determinant of HRQL in patients with CAD [5] and following CABG [18]. However, this association cannot be examined without taking into account several clinical factors. Although age is an important risk factor for both CAD [19] and cognitive decline [6], it is uncertain whether it is an independent factor for predicting HRQL [20]**.** Evidence also suggests that depressive mood and fatigue are associated with both cognitive complaints and poor outcome in patients with CAD, including decreased HRQL [4, 21].

Because there is a paucity of studies on long-term perceived cognitive functioning after PCI, the current study examined perceived cognitive functioning immediately after PCI and at 1-year follow-up, and its association with HRQL. We also examined whether symptoms of depression and fatigue are associated with changes in perceived cognition and HRQL, and may (partially) explain the link between perceived cognition and HRQL. Finally, we examined whether age is associated with changes in perceived cognition and HRQL [6].

## Methods

### Patient Population and Procedure—THORESCI Study

The current study was part of an ongoing prospective cohort study, **T**ilburg **H**ealth **O**utcomes **R**egistry of **E**motional **S**tress after **C**oronary **I**ntervention (THORESCI), which recruits participants from the clinical standard of care PCI Registry at the Elisabeth-TweeSteden Hospital in Tilburg, the Netherlands. All patients scheduled for elective or acute PCI for ≥1 coronary occlusions were included, provided that patients were aged ≥18 and had sufficient understanding of the Dutch language to fill out questionnaires. Medical records were checked to see whether patients had a life threatening comorbidity (e.g., metastasized cancer) or cognitive disorder (i.e., dementia or Alzheimer’s), and if so, they were excluded. On the day of the PCI, patients were approached by a member of the research team who explained the study content and its requirements. After giving written consent, the patients were asked to fill out a psychosocial survey at home, between 0 and 5 days after PCI (inclusion (baseline)), and at 1, and 12 months post-PCI by e-mail or on paper. The study protocol is in keeping with the Helsinki declaration and was approved by the institutional medical ethics review board.

### Measures

#### Demographics

Age, gender, and education level were obtained from self-report questionnaires at inclusion (T0). Educational level was recoded into a dichotomous variable—high education (at least high school) vs. low education.

#### Perceived Cognitive Functioning

*Perceived concentration* was measured with dedicated questions. From the Patient Health Questionnaire (PHQ-9) [22], the question asking to report on diminished ability to think or concentrate, or more indecisiveness [23], i.e., “*Trouble concentrating on things, such as reading the newspaper or watching television?,”* was used. This item was rated on a Likert scale from 0 (“not at all”) to 3 (“almost every day”) [22]. From the World Health Organization Quality of Life assessment instrument (WHOQOL-Bref) [24], we used *“How well are you able to concentrate”* to assess perceived concentration. This item was rated on a Likert scale from 1 (“not at all”) to 5 (“extremely”) [24]. We excluded the item from the quality of life assessment (see below). The scores of this item were reversed for this study for it to correspond with the scores of the PHQ-9. To calculate the total perceived concentration score, these two questions were standardized and summed to generate a total concentration score, with a higher score indicating worse concentration. The range for the standardized scores was on a Likert scale from −1 to 3, with a mean of 0. Internal consistency in this study was acceptable (Cronbach’s alpha Baseline = 0.67; 1 month = 0.81; 12 months = 0.80).

*Perceived attention* was measured with the facet “acting with awareness” from the short form of the Five Facet Mindfulness Questionnaire (TFMQ-SF); e.g., “*I do jobs or tasks automatically, without being aware of what I am doing”; “I find myself doing things without paying attention”* [25]**.** Items were scored on a 5-point Likert-type scale ranging from 1 (“never or rarely true”) to 5 (“very often or always true”) [25], with higher scores indicating worse attention. Total score was calculated by computing the mean of the scores on the five items per measurement moment. In this study, internal consistency was good (Cronbach’s alpha Baseline = 0.77; 1 month = 0.80; 12 months = 0.84).

#### Health-Related Quality of Life

The World Health Organization Quality of Life assessment instrument (WHOQOL-Bref), a reliable and valid questionnaire, was used to measure HRQL [24]. Its 26 items are rated on a Likert scale from 1 (“very poor/not at all/very dissatisfied/never”) to 5 (“very good/extremely/very satisfied/always”) that represent one facet (“General health and overall quality of life”), and four domains (“Physical health”, “Psychological health”, “Social relationships”, and “Environment”). In the present study, question 7 *“How well are you able to concentrate”*, was removed from the score of its domain (“Psychological health”) because it is already used to measure perceived concentration. The scores on the other domains are unaffected by this action.

#### Depressive Symptoms

The Beck Depression Inventory (BDI) was used to measure somatic-affective and cognitive-affective symptoms of depression [26]**.** The BDI consists of 21 items with 4 scores per item ranging from“normal” to “severe depressed” state [26]. Questions in this instrument refer to the past week. In this study, internal consistency was good (Cronbach’s alpha = 0.87).

#### Fatigue

Fatigue was assessed with fatigue subscale of the 24-item Health Complaints Scale (HCS). This questionnaire has high internal consistency (Cronbach’s α > 0.89), adequate test-retest reliability (*r* > 0.69), and good construct validity [27]. The fatigue subscale consisted of four items, which were summed, with higher scores indicating more fatigue complaints. In the current dataset, Cronbach’s alpha was >0.80. All items are rated on a Likert scale from 0 (“not at all”) to 4 (“a lot”).

#### Medical Covariates

At inclusion, the patient’s medical records were used to obtain information on the indication for PCI. PCI as invasive treatment of acute coronary syndrome were compared to patients with elective PCI.

Cardiac history (heart failure, previous MI, previous CABG, single vs. multi-vessel disease), medication use (antidepressants, beta-blockers, antiplatelet drugs, anticoagulant drugs, ACE inhibitors, calcium antagonists), and risk factors (family history of CAD, hypercholesterolemia, and diabetes mellitus type 2) were taken from the patient’s medical records at inclusion (T0).

Patients received local sedation (1–2% lidocaine) during the procedure.

### Statistical Analysis

#### Baseline Characteristics

First, we calculated median splits for the longitudinal person-mean of the concentration and attention problems scores for presentation purposes and to examine baseline differences in patient characteristics between patients with and without perceived concentration or attention complaints. If patients were exactly at the median, they were included as low attention/concentration complaints. Student’s *t* tests were then done in case of continuous variables, and chi-square tests in case of categorized variables to compare the groups.

#### HRQL Change over Time

To assess whether HRQL changed significantly over time, time was included as the only predictor in the first step of the general linear mixed modeling analysis (see explanation below). To compare low vs. high concentration/attention groups in terms of HRQL change over time, the mean scores and standard deviations of the groups on HRQL in the three time periods were calculated.

#### General Linear Mixed Modeling Analysis

General linear mixed modeling analysis was performed to examine the effects of the level and change of perceived cognition over time on the course of HRQL over the 12-month follow-up period, using maximum likelihood estimation, and an unstructured covariance matrix with a two-level structure (i.e., repeated measurement occasions (lower level), participant (higher level)). This technique is suitable for analysis of repeated measurements, as it takes the possibility of correlated data into account. In addition, in contrast to traditional repeated measures ANOVA, one missing measurement occasion does not automatically lead to exclusion of that patient from analysis, limiting bias and preserving statistical power. Another advantage to linear mixed modeling analysis is the possibility of measuring variables as fixed variables or as time-varying variables.

Person—means of both perceived concentration and attention complaints scores over the three included measurement occasions were calculated first (between-subjects effects), and then the deviation from this person—mean at each time point was calculated in order to get information on the person-specific change in perceived concentration and attention complaints (within subjects effects). These continuous variables constituted our independent variables. Five separate analyses were done for each HRQL domain, and analyses were performed separate for perceived concentration and perceived attention. In step 1, the two concentration complaints variables (person—mean and deviation) were entered (*Unadjusted model*). In step 2, the models were adjusted for demographics (gender, education) and medical covariates (PCI indication, cardiac history, antidepressant use). In the third step, we added depressive symptoms and fatigue, and finally age was added to the model. The same steps were taken for perceived attention complaints scores. All demographic variables and medical covariates were included as fixed variables. Depressive symptoms and fatigue were added as time-varying variables. All statistical analyses were performed using SPSS 22 (IBM SPSS Statistics for Windows, Version 22. Armonk, NY: IBM Corps USA). To prevent Type I error arising from multiple testing, a *p* value of *p* < .02 was considered significant.

## Results

### Sample Characteristics

In this ongoing cohort study, a subsample of the THORESCI study was used in which all participants were required to have filled out the WHOQOL-Bref and/or PHQ-9 and TFMQ-SF in order to be included in the final analysis set. Therefore, based on the data extraction of February 2016, we included 384 Baseline assessments, 354 1-month assessments, and 233 12-months assessments in our analyses (*M*_age_ 63.44, SD 10.69 years, 79.0% Men). The considerably lower number of participants assessed at 12 months is explained by the current study being an ongoing cohort study. The statistical technique we chose is robust for such a decline though. A total of 17.2% of participants included at baseline had dropped out at 12-month follow-up (*n* = 55), or had deceased within that timeframe (*n* = 10). In comparing the included sample with the dropouts/deceased sample on baseline characteristics, results showed that the dropouts/deceased were older (*p* = .006), more often had heart failure (*p* = .038), and reported higher scores of depression (*p* = .033).

Baseline sample characteristics are presented in Table 1. With respect to demographic characteristics, patients with above median scores of total perceived concentration complaints (*n* = 190) were significantly younger (*p* = .034). Patients with perceived concentration complaints were more often on anti-depressants (*p* = .024) and more often underwent an acute PCI (*p* = .019). In addition, patients with perceived concentration and attention complaints reported higher scores of depression (*p* < .001) and fatigue (*p* < .001).

**Table 1:** Baseline patient characteristics

	Total *n* = 384				Total *n* = 376^a^			
	Low concentration problems score (*n* = 194)	High concentration problems score (*n* = 190)	Test statistic	*p* value	Low attention problems score (*n* = 196)	High attention problems score (*n* = 180)	Test statistic	*p* value
Demographics								
Sex (male)	79.9% (155)	77.9% (148)	0.23	.631	82.1% (161)	76.1% (137)	2.08	.150
Age mean (SD)	64.63 (8.99)	62.53 (10.33)	4.53	**.034**	64.09 (9.22)	62.94 (10.12)	1.34	.248
High education (≥8 years)^b^	69.2% (126)	67.4% (122)	2.07	.355	64.8% (118)	72.7% (128)	5.25	.*072*
Medical								
Cardiac history^c^								
Heart failure	2.7% (5)	2.1% (4)	0.12	.730	2.1% (4)	2.3% (4)	0.02	.900
MI	15.1% (28)	21.9% (41)	2.92	.*087*	15.7% (30)	20.0% (35)	1.15	.283
CABG	7.0% (13)	8.0% (15)	0.14	.705	7.9% (15)	6.9% (12)	0.13	.716
Risk factors								
Genetic risk^c^	40.5% (75)	43.9% (82)	1.05	.518	38.2% (73)	47.1% (82)	2.96	.*086*
Hypercholesterolemia^c^	36.8% (68)	36.9% (69)	0.00	.977	35.6% (68)	38.5% (67)	0.33	.566
Diabetes mellitus type 2	13.4% (26)	11.1% (21)	0.49	.482	14.3% (28)	8.9% (16)	2.65	.104
Medication use^d^								
Antidepressants	3.2% (6)	8.6% (16)	4.77	**.024**	5.8% (11)	5.1% (9)	0.07	.796
Beta-blockers	33.9% (63)	38.5% (72)	0.87	.352	34.6% (66)	37.1% (65)	0.27	.606
Antiplatelet drugs	43.0% (80)	42.8% (80)	0.00	.964	41.9% (80)	43.4% (76)	0.09	.765
Anticoagulant drugs	5.9% (11)	7.0% (13)	0.17	.683	7.9% (15)	5.1% (9)	1.10	.295
ACE inhibitors	34.4% (64)	40.6% (76)	1.55	.214	35.1% (67)	39.4% (69)	0.74	.390
Calcium antagonists	14.0% (26)	15.5% (29)	0.17	.677	16.2% (31)	12.0% (21)	1.34	.247
PCI								
Acute	60.8% (118)	72.1% (137)	5.48	**.019**	65.3% (128)	68.9% (124)	0.55	.460
Multi-vessel treatment	22.3% (43)	17.9% (34)	1.15	.284	20.5% (40)	19.4% (35)	0.07	.796
Psychological characteristics								
Depressive mood mean (SD)	7.19 (4.33)	11.35 (7.33)	43.10	**<.001**	7.53 (4.39)	11.16 (7.50)	31.38	**<.001**
Fatigue mean (SD)	3.96 (3.65)	6.35 (4.56)	29.93	**<.001**	4.19 (3.92)	6.08 (4.45)	17.85	**<.001**

Bold *p* values indicate statistical significance, italic *p* values indicate significance on a trend level

^a^missing 8 participants due to missing attention scores

^b^missing 21 participants

^c^missing 12 participants

^d^missing 10 participants

### Change of Health Related Quality of Life over Time

As the initial analysis, General health and overall QoL improved significantly over the 12-month follow-up period (*F* = 9.80, *p* < .001) (first table line, Table 2). General health and overall QoL did not improve at 1 month (*p* = .222), but significantly improved at 12 months compared to baseline (*p* < .001) and 1-month follow-up (*p* < .001). The physical subdomain score also showed a main effect of time (*F* = 23.33, *p* < .001). Physical health improved significantly at 1-month follow-up (*p* < .001) and 12-month follow-up (*p* < .001), compared to baseline. A significant improvement between 1-month follow-up and 12-month follow-up was also found (*p* = .03). Social relationship scores showed a main effect of time as well (*F* = 3.13, *p* = .045). Between baseline and 1-month follow-up, no significant improvement was found (*p* = .921); however, between baseline/1 month and 12-month follow-up, a significant decline was found (*p* = .029, *p* = .016). Psychological health and environment scores showed no main effect of time (*F* = 1.88, *p* = .155; and *F* = .165, *p* = .848 respectively).

**Table 2:** Results from multivariable linear mixed models for concentration problems total score

	General health and overall QoL			Physical health			Psychological health^a^			Social relationships			Environment		
Concentration problems score	Est.	SEM	*p*	Est.	SEM	*p*	Est.	SEM	*p*	Est.	SEM	*p*	Est.	SEM	*p*
Time (baseline, 1 year)	0.44	.088	**<.001**	1.00	.144	**<.001**	−0.07	.123	.581	−0.29	.172	*.091*	0.13	.121	.269
Step 1. Unadjusted model															
Time (baseline, 1 year)	0.37	.111	**<.001**	1.00	.140	**<.001**	−0.07	.123	.596	−0.28	.174	.114	0.13	.122	.280
Concentration between	−0.45	.042	**<.001**	−0.79	.069	**<.001**	−0.78	.062	**<.001**	−0.65	.074	**<.001**	−0.58	.067	**<.001**
Concentration within	−0.12	.031	**<.001**	−0.33	.053	**<.001**	−0.20	.044	**<.001**	−0.13	.058	*.028*	−0.12	.041	**.003**
Step 2. Adjusted for demographic and clinical variables															
Time (baseline, 1 year)	0.40	.091	**<.001**	0.94	.149	**<.001**	−0.14	.129	.266	−0.40	.181	*.029*	0.06	.129	.630
Concentration between	−0.45	.045	**<.001**	−0.76	.074	**<.001**	−0.78	.068	**<.001**	−0.65	.079	**<.001**	−0.60	.071	**<.001**
Concentration within	−0.13	.032	**<.001**	−0.32	.055	**<.001**	−0.21	.045	**<.001**	−0.15	.060	**.014**	−0.13	.043	**.002**
High education	0.50	.924	.592	0.44	.247	.*078*	0.19	.227	.394	0.40	.262	.130	1.05	.234	**<.001**
Male gender	0.17	.173	.328	0.89	.289	**.002**	0.54	.265	*.043*	−0.40	.305	.188	0.16	.273	.561
Acute PCI	0.10	.145	.476	0.39	.242	.108	0.16	.223	.479	0.82	.260	**.002**	0.09	.230	.695
Cardiac history	−0.18	.159	.251	−0.22	.264	.398	−0.00	.242	.994	−0.11	.280	.704	−0.23	.250	.352
Antidepressant use	−0.38	.325	.248	−1.03	.539	*.057*	−0.98	.497	*.050*	−0.45	.574	.430	0.13	.512	.804
Step 3. Adjusted for psychological variables (clinical + psychological variables)															
Concentration between	−0.18	.041	**<.001**	−0.29	.064	**<.001**	−0.41	.063	**<.001**	−0.35	.081	**<.001**	−0.38	.071	**<.001**
Concentration within	−0.08	.030	**.009**	−0.22	.051	**<.001**	−0.13	.043	**.002**	−0.08	.060	.155	−0.09	.043	*.041*
Mood	−0.10	.009	**<.001**	−0.12	.014	**<.001**	−0.15	.013	**<.001**	−0.13	.017	**<.001**	−0.09	.014	**<.001**
Fatigue	−0.08	.012	**<.001**	−0.23	.020	**<.001**	−0.06	.018	**.001**	−0.04	.024	.102	−0.04	.019	*.022*
Step 4. Complete model (clinical + psychological variables + age)															
Concentration between	−0.16	.041	**<.001**	−0.30	.065	**<.001**	−0.39	.064	**<.001**	−0.35	.082	**<.001**	−0.35	.071	**<.001**
Concentration within	−0.08	.030	**.009**	−0.23	.051	**<.001**	−0.13	.043	**.003**	−0.08	.060	.155	−0.09	.043	*.045*
Age	0.02	.006	**.007**	−0.01	.010	.251	0.03	.009	**.004**	0.00	.012	.873	0.03	.011	**.006**

Bold *p* values indicate statistical significance, italic *p* values indicate significance on a trend level

*PCI* percutaneous coronary intervention, *QoL* quality of life, *Est*, estimate, *SEM* standard error of the mean, ^a^Score without concentration item

Because concentration and attention were only moderately correlated (*r* = 0.30), further analyses were performed separately for perceived concentration complaints scores and attention complaints.

**Fig. 1:**
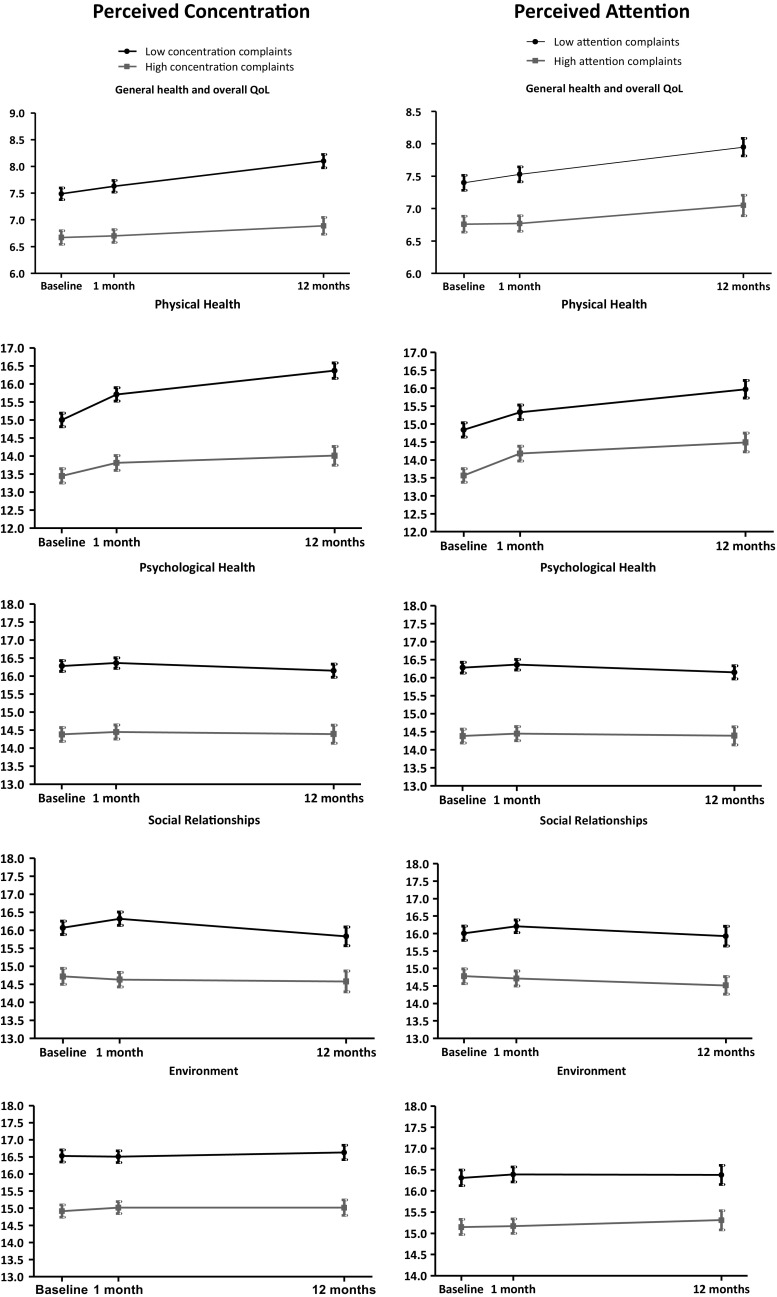
Health related quality of life over time. Higher WHOQOL-Bref scores denote better quality of life; higher perceived concentration/attention scores denote more complaints. *QoL* quality of life

Figure 1 shows that the mean scores of all the HRQL domains over time for low concentration/attention complaints are higher than high concentration/attention complaints. In addition, scores on general health and overall QoL and the physical domain increase over time in all groups, while HRQL scores on other domains remain stable over time (Fig. 1).

### Concentration and HRQL

Results showed a main effect of between-subject differences in perceived concentration complaints on general health and overall QoL (*F* = 118.22, *p* < .001), and of within-subject differences in perceived concentration complaints scores as well (*F* = 15.19, *p* < .001). These main effects of between- and within-subject differences in concentration were similar for physical health (*F* = 129.72, *p* < .001; *F* = 40.31, *p* < .001), psychological health (*F* = 160.10, *p* < .001; *F* = 20.63, *p* = .001), and environment (*F* = 74.88, *p* < .001; *F* = 9.11, *p* = .003). For social relationships, the between-subject differences in concentration were significant (*F* = 77.30, *p* < .001); however, the within-subject differences were found with a significance level between *p* = .01 and *p* = .05. Estimates are shown in Table 2, which represents the unstandardized regression coefficients of the dependent variable when the independent variable increases with one unit.

In unadjusted analyses, better perceived concentration was associated with better general health and overall QoL over time (*p* < .001), for between- as well as within-subject differences. Similar effects remained when adjusting for demographic and clinical covariates. None of the demographic or clinical covariates were associated with general health and overall QoL across time. A substantial part of the relation between perceived concentration complaints and HRQL was explained by depressive mood (*p* < .001) and fatigue (*p* < .001; Step 3); however, results showed that increased perceived concentration complaints remained a predictor for worse general health and overall QoL. The same results showed when age (*p* = .007) was added to the model in a final step.

Between- and within-subject differences in perceived concentration complaints were also related to differences in physical health over time, which remained the same in the adjusted analyses. Male gender was associated with better physical health, and antidepressant use tended to be associated with poorer physical health. Based on the reduction in estimates in step 3, depressive mood and fatigue only partially explained the relation between perceived concentration complaints and physical health. Age did not contribute to this association.

Between- and within-subject differences in concentration complaints were associated with psychological health, social relationships, and environment. These effects remained in the adjusted analyses. In the third step, it was shown that higher depressed mood was strongly related to poorer psychological health, social relationships, and environment, but fatigue was not associated with social relationships (*p* = .102) or environment (*p* = .022). However, with respect to between-subject differences, more perceived concentration complaints remained a predictor of poorer HRQL in the three domains, even though strong reductions in estimates were observed. Within-subject changes were only significantly related to psychological health and environment after controlling for psychological factors. Older age was associated with better psychological health, while age did not affect social relationships. Again, more perceived concentration complaints remained a predictor of poorer HRQL in the three domains.

### Attention and HRQL

There was a significant between-subjects effect of perceived attention complaints (*F* = 46.86, *p* < .001) and a significant within-subjects effect (*F* = 8.51, *p* = .004), both reflecting increased attention complaints being associated with poorer HRQL (Table 3). For the domain scores, this main between-subjects effect of perceived attention complaints was also found (physical *F* = 47.22, *p* < .001; psychological *F* = 71.91, *p* < .001; social *F* = 44.67, *p* < .001; environment *F* = 37.87, *p* < .001). The main within-subjects effect of perceived attention complaints was only found for the physical component (*F* = 5.58, *p* = .018), indicating that physical health changes within a person covaries with changes in perceived attention complaints over time.

**Table 3:** Results from multivariable linear mixed models for attention problems total score

Attention problems scores	General health and overall QoL			Physical health			Psychological health^a^			Social relationships			Environment		
	Est.	SEM	*p*	Est.	SEM	*p*	Est.	SEM	*p*	Est.	SEM	*p*	Est.	SEM	*p*
*Step 1. Unadjusted model*															
Time (baseline, 1 year)	0.39	.089	**<.001**	0.99	.147	**<.001**	−0.08	.127	.554	−0.25	.175	.147	0.15	.124	.234
Attention between	−0.16	.023	**<.001**	−0.27	.039	**<.001**	−0.30	.035	**<.001**	−0.26	.039	**<.001**	−0.22	.036	**<.001**
Attention within	−0.05	.016	**.004**	−0.06	.026	**.018**	−0.03	.022	.160	−0.01	.030	.770	−0.01	.021	.655
Step 2. Adjusted for demographic and clinical variables															
Time (baseline, 1 year)	0.37	.093	**<.001**	0.94	.156	**<.001**	−0.16	.133	.237	−0.39	.184	*.036*	0.09	.132	.483
Attention between	−0.15	.024	**<.001**	−0.25	.040	**<.001**	−0.29	.036	**<.001**	−0.28	.040	**<.001**	−0.23	.036	**<.001**
Attention within	−0.06	.017	**<.001**	−0.07	.029	**.012**	−0.04	.024	*.*122	−0.01	.032	.675	−0.00	.023	.894
High education	0.28	.160	.*080*	0.51	.296	*.058*	0.33	.247	.178	0.50	.271	*.066*	1.16	.245	**<.001**
Male gender	0.11	.187	.565	0.67	.313	*.033*	0.31	.287	.278	−0.72	.316	*.024*	−0.03	.286	.923
Acute PCI	0.04	.155	.773	0.22	.261	.406	0.02	.239	.947	0.64	.263	**.016**	−0.05	.238	.831
Cardiac history	−0.33	.169	*.055*	−0.40	.284	.160	−0.29	.260	.272	−0.33	.286	.255	−0.47	.259	.*073*
Antidepressant use	−1.07	.334	**.001**	−2.21	.563	**<.001**	−2.19	.516	**<.001**	−1.47	.566	**.010**	−0.81	.513	.114
Step 3. Adjusted for psychological variables (clinical + psychological variables)															
Attention between	−0.04	.019	*.028*	−0.07	.031	*.028*	−0.14	.030	**<.001**	−0.16	.038	**<.001**	−0.14	.034	**<.001**
Attention within	−0.04	.016	**.011**	−0.03	.030	.193	0.00	.023	.963	0.02	.031	.576	0.02	.023	.419
Mood	−0.11	.009	**<.001**	−0.14	.015	**<.001**	−0.17	.013	**<.001**	−0.14	.017	**<.001**	−0.10	.014	**<.001**
Fatigue	−0.09	.012	**<.001**	−0.25	.021	**<.001**	−0.07	.018	**<.001**	−0.04	.024	*.076*	−0.06	.019	**.003**
Step 4. Complete model (clinical + psychological variables + age)															
Attention between	−0.04	.019	*.038*	−0.07	.031	*.026*	−0.13	.030	**.001**	−0.16	.038	**<.001**	−0.13	.033	**<.001**
Attention within	−0.04	.016	**.013**	−0.03	.026	.190	−0.00	.023	.908	0.02	.038	.567	0.02	.023	.380
Age	0.02	.006	**.006**	−0.00	.010	.606	0.03	.009	**.001**	0.01	.012	.524	0.04	.011	**.001**

Bold *p* values indicate statistical significance, italic *p* values indicate significance on a trend level

*PCI* percutaneous coronary intervention, *QoL* quality of life, *Est*. estimate, *SEM* standard error of the mean

^a^Score without concentration item

When adding clinical and demographic covariates, the relation between attention and general health and overall QoL remained similar. In step 3, both depressive symptoms and fatigue were strongly related to general health and overall QoL (*p* < .001), and may partially explain this effect, based on the reduction of the attention complaints estimate and the reduction of the significance level (*p* = .028). Older age was significantly associated with better general health and overall QoL (*p* = .006), and further reduced the association between attention and general health and overall QoL.

Between-subjects differences in perceived attention complaints were significantly related to differences in all HRQL domains, and within-subjects differences in perceived attention complaints were only significantly related to differences in physical health over time. In adjusted analyses, these effects remained in the psychological, social relationship, and environment domains. Depressive symptoms were associated with poorer HRQL in all domains, and fatigue with poorer physical health, psychological health and environment, but not social relationships. Attention remained a predictor for psychological health, social relationships, and environment when psychological variables were added; however, reduction in the estimate indicates that mood and fatigue may partially explain this effect. Older age was related to better psychological health and environment, but no association was found with physical health and social relationships.

## Discussion

Results of the current study indicate that between-subject differences and within-subject changes in perceived concentration and attention in PCI patients over the first year post-PCI were strongly associated with poorer HRQL during this time period, independent of demographic and clinical covariates. Contrary to between-subject differences in perceived cognition, which were associated with all HRQL domains, within-subject changes in perceived attention were not associated with psychological health, social relationships, and environment over time. Depression and fatigue only partially accounted for the longitudinal association between perceived cognitive function and HRQL.

With respect to HRQL, the domains general health and overall QoL and physical health improved over time in this sample of PCI patients. This is in line with previous research validating the benefit of PCI in CAD patients [1, 2]. However, the scores of the domain social relationships tended to decrease in this population over time, indicating that 12 months after undergoing a PCI, CAD patients may perceive less social support.

Perceived cognitive complaints were associated with a decline in multiple areas of HRQL, even after controlling for factors that are known to be related to HRQL, which is in line with a previous study in CABG patients [18]. This indicates also that using a PCI control group to assess cognitive changes after invasive cardiovascular treatment [3, 11, 13, 15, 18] may be biasing the results, as the PCI group may be experiencing complaints themselves. In contrast to other studies on the topic, our study investigated both between- and within-subjects effects. The strong association between the poorer person-mean scores of participants on perceived cognition and poorer HRQL might be explained by pre-existing cerebrovascular abnormalities, such as white matter hyperintensities and infarct-like lesions, which are common in CAD patients, and associated with poorer cognitive functioning [8, 9]. Future studies including, e.g., magnetic resonance imaging (MRI) might be able to examine this explanation further. In addition, it is of importance that perceived cognitive complaints are included as an outcome variable in future studies, because perceived cognitive complaints might predict the progression of imminent cognitive decline [17]. Cognitive decline (i.e., the within-subject change) is associated with less ability to engage in activities of daily living [18], which may explain the association with a decline in HRQL. Several core executive functions seem relevant for mental as well as physical HRQL, due to the everyday life activities people undertake in which cognitive functioning is needed (e.g., switching between tasks) [28]. When these cognitive functions are limited, there is less ability to engage in activities of daily living, thereby negatively affecting HRQL [18]. Moreover, the independent relationship between mood, fatigue, and HRQL [29] might be explained by limitations in daily functioning as well.

One important observed difference between attention and concentration complaints was that changes in perceived attention complaints within a person did not relate to changes in psychological health, social relationships or environment, while within-subject differences in concentration complaints were associated with all HRQL domains. An explanation might be sought in differences in patient characteristics. Patients with high perceived concentration complaints were more likely to have undergone an acute PCI. In these acute cases, patients were either in danger of cardiac arrest or were experiencing myocardial infarction preceding the intervention. Cardiac arrest might lead to oxygen deficiency in the brain, a well-known cause of decline in cognitive functioning [30]. Therefore, we might hypothesize that perceived attentional complaints may have a different underlying cause than perceived concentration complaints. For example, ruminative trains of thought leading to disengagement of attention from the momentary task or experience toward personally relevant and negative material [31], may cause perceived attention complaints rather than oxygen deficiency in the brain would do. This would deserve further investigation in future studies. The relation with poorer HRQL may be explained by disengagement of attention and rumination being associated with reduced mindfulness and well-being [32]. As a consequence of the different results found between acute PCI and elective PCI in relation to attention and concentration complaints, we suggest that a differentiation between acute PCI and elective PCI is warranted in future studies.

Depressive symptoms and fatigue partly accounted for the effects of perceived cognitive complaints for all HRQL domains although effects remained significant. Depressed mood and fatigue have been associated with both CAD and cognition [21, 23, 33]. Not only does depressed mood increase the risk of developing CAD [34], but both depressed mood and fatigue are common in patients with CAD, which in turn is associated with worsened outcome in CAD patients, including decreased HRQL [21, 33–35]. Our results are in line with prior findings showing perceived decline in cognitive functioning to be higher in the subjects with higher depression scores and lower ratings of subjective health [36], but also clearly show the partial independence of perceived cognitive function from these variables. Depressive symptoms and fatigue remain important variables to take into consideration when exploring the relationship between cognitive functioning and poor HRQL.

Our study suggests that older age is associated with better general health and overall QoL, psychological health, and a better environment. However, it hardly interfered with the association between perceived cognition and HRQL, as worse perceived cognition remained a predictor of poorer HRQL. However, there seems to be an interaction, with the average age being higher in the low perceived cognition complaints group than the high perceived cognition complaints group. It is possible that the greater burden of illness in participants of working age has a more negative outcome on perceived cognitive functioning in younger than in older study participants. Older participants might perceive less cognitive change, due to retirement and might therefore be less exposed to higher cognitively demanding tasks. Evidence also suggests that middle-aged CAD patients are more affected by emotional distress than older-aged patients [37]. This is in line with the current results showing that older age is associated with better general health and overall QoL, psychological health, and environment.

In a comparable study in CABG patients with similar results, cognition was assessed objectively using neuropsychological tests [18]. This is a frequently used method for assessing cognition to detect impairments [6]. In our study, participants reported on how they perceived attention and concentration. Some changes in cognition might not be measured through objective tests, but still may be perceived by the patient. Another advantage for assessing perceived cognition is that there is no sign of practice effects, which often happens with objective tests that are repeated over time [38]. However, further study is needed to examine whether and which other components of cognition serve as potential predictors of HRQL, and standardized neuropsychological tests might help to further investigate this. In addition, it might be interesting to study PCI patients in a longitudinal study in which standardized neuropsychological tests are used to assess cognition in order to see whether the same relationship is found between objectively assessed cognition and HRQL.

From a clinical perspective, results may suggest that perceived cognitive functioning and HRQL have had time to improve in 1 year. The strong correlation of change in perceived cognitive functioning with change in HRQL supports the importance of addressing cognitive function when evaluating recovery from PCI. In addition, clinicians should evaluate cognition and consider including neurocognitive screening as part of treatment after PCI, for example in cardiac rehabilitation [39].

There are several limitations that should be taken into consideration. First, cognition was only assessed by means of self-report, which may be subjected to systematic biases and may not reflect neuropsychological dysfunction [36]. Perceived cognition therefore may not give estimates of actual cognitive skills, and lower cognition scores in the current study cannot be interpreted as a decline in cognition, but rather how patients perceive how their cognition has changed. Subjectively measuring cognitive functioning might therefore be more associated with other processes, such as depressive mood, than objective assessment. Previous findings of Vingerhoets et al. reported that high reported depression scores had no effect on cognitive performance but did affect patients who subjectively felt that their cognitive abilities were decreased [40]. Second, perceived concentration and attention complaints were not assessed through an existing questionnaire, but through combining items and facets from existing questionnaires. The choice for the TFMQ-SF acting with awareness facet was made because of its strong association with executive functioning [41]. Further, by using the item for concentration from the WHOQOL-bref domain“psychological health,” the items for assessing concentration might be highly correlated with this domain. In addition, this domain is therefore not comparable to scores on this domain in other studies. Also, it is impossible to know from this study whether these findings are unique for post-PCI patients, due to the absence of a comparison group. Moreover, the majority of our sample was treated acutely, making it impossible to measure pre-PCI perceived cognition, and therefore, it is impossible to know if patients’ self-perceived cognition was any different prior to PCI. Because this is a single-center study in PCI patients only, the generalizability of the results to other PCI or other cardiac populations remains unknown. Finally, 17% of the sample had dropped out or were deceased at the 12-month measurement occasion, due to older age, heart failure, and increased baseline depression. This might have affected the results.

Strengths of this study were the relatively large sample size, the longitudinal and multivariable design with time-varying predictors, and co-variates. Previous studies have mentioned the importance of including emotional factors [5, 42], so the inclusion of psychological variables was another substantial strength. Also, the current study was the first study to include PCI patients as main group to investigate the relationship between cognition and HRQL over time. Further, our study distinguished between- and within-subjects effects, which makes this the first study to examine whether changes within a person were associated with changes in HRQL. Finally, the Elisabeth-TweeSteden hospital is one of the largest PCI centers in the Netherlands, serving a cardiac population that is representative of the cardiac population in the Netherlands.

To conclude, between-subject differences and within-subject changes in perceived concentration and attention in PCI patients were strongly associated with HRQL over time, such that poorer perceived concentration and attention were associated with poorer HRQL, independent of demographic, and clinical variables. Mood and fatigue seemed to partially account for these associations. Age did not interfere with the association between perceived cognition and HRQL. Further study is warranted on objective cognitive assessment in PCI patients and the role of cognitive functioning on HRQL. We would recommend clinicians to take cognitive functioning into consideration as part of treatment and rehabilitation after PCI.
